# Supplementary material for: MetaRibo-Seq measures translation in microbiomes
Source: Nat Commun. 2020 Jun 29;11:3268. doi: 10.1038/s41467-020-17081-z (PMC7324362; doi:10.1038/s41467-020-17081-z)
Supplement: Supplementary file 10 — Supplementary Data 7 [file 41467_2020_17081_MOESM10_ESM.zip › File2/Confidence_VeryHigh_Taxonomy/85310_out.krona.html]

Javascript must be enabled to view this page.

members
magnitude
magnitudeUnassigned
count
unassigned
taxon
rank

85310\_out

46

2
superkingdom
46

46
1239
phylum

46
186801
class

3
186802
order
46

SRS015190\_contig\_number\_12921SRS050925\_contig\_number\_8735SRS056519\_contig\_number\_12982

31979
family
1

genus
1485
1

species
1262785
1

SRS051031\_contig\_number\_13467

family
186803
3

28050
genus
3


SRS019161\_contig\_number\_36435SRS043667\_contig\_number\_5905SRS044535\_contig\_number\_2242
3
species
28052

family
186806
39


SRS144183\_contig\_number\_36089
1
genus
1730
39


SRS013951\_contig\_number\_26869SRS014855\_contig\_number\_6780SRS018984\_contig\_number\_28995SRS043701\_contig\_number\_17117SRS052027\_contig\_number\_18727SRS065504\_contig\_number\_19795SRS100021\_contig\_number\_4549SRS1055022\_contig\_number\_3489SRS143342\_contig\_number\_21194SRS143780\_contig\_number\_27221SRS146813\_contig\_number\_9066SRS148091\_contig\_number\_11083
12
species
142586

species
1897026

SRS014235\_contig\_number\_45309SRS015663\_contig\_number\_27463SRS058723\_contig\_number\_5910SRS077335\_contig\_number\_7729SRS098827\_contig\_number\_12611SRS104311\_contig\_number\_32788SRS147271\_contig\_number\_32489SRS147377\_contig\_number\_contig-100\_756.42209
8

1

SRS063040\_contig\_number\_contig-100\_137.256210
species
1262889


SRS017191\_contig\_number\_18427
1
39485
species

13

SRS015431\_contig\_number\_84546SRS015578\_contig\_number\_33045SRS019267\_contig\_number\_contig-100\_181.52995SRS024331\_contig\_number\_contig-100\_1111.181303SRS047044\_contig\_number\_17793SRS063985\_contig\_number\_15618SRS075878\_contig\_number\_5457SRS077502\_contig\_number\_1831SRS078176\_contig\_number\_contig-100\_986.101604SRS144135\_contig\_number\_28771SRS146812\_contig\_number\_50108SRS148159\_contig\_number\_3192SRS148253\_contig\_number\_contig-100\_225.46250
1262892
species

species
1263077
3

SRS014979\_contig\_number\_986SRS023346\_contig\_number\_6813SRS147919\_contig\_number\_18236
